# MnTBAP therapy attenuates the downregulation of sodium transporters in obstructive kidney disease

**DOI:** 10.18632/oncotarget.23037

**Published:** 2017-12-07

**Authors:** Mi Liu, Yangyang Zhu, Ying Sun, Zhaoying Wen, Songming Huang, Guixia Ding, Aihua Zhang, Zhanjun Jia, Yue Zhang

**Affiliations:** ^1^ Nanjing Key Laboratory of Pediatrics, Children’s Hospital of Nanjing Medical University, Nanjing, China; ^2^ Department of Cardiology, Sun Yat-sen Memorial Hospital, Sun Yat-sen University, Guangzhou, China; ^3^ Department of Nephrology, Children’s Hospital of Nanjing Medical University, Nanjing, China; ^4^ Jiangsu Key Laboratory of New Drug Research and Clinical Pharmacy, Xuzhou Medical University, Xuzhou, China; ^5^ Department of Radiology, Beijing Anzhen Hospital, Capital Medical University, Beijing Institute of Heart, Lung and Blood Vessel Diseases, Beijing, China

**Keywords:** unilateral ureteral obstruction, mitochondrial oxidative stress, sodium transporters, MnTBAP, SOD

## Abstract

Ureteral obstruction is associated with reduced expressions of renal sodium transporters, which contributes to impaired urinary concentrating capacity. In this study, we employed a synthetic mitochondrial superoxide dismutase 2 (SOD2) mimic MnTBAP to investigate the role of mitochondrial oxidative stress in modulating the sodium transporters in obstructive kidney disease. Following unilateral ureteral obstruction (UUO) for 7 days, a global reduction of sodium transporters including NHE3, NCC, NKCC2, and ENaCα was observed as determined by qRT-PCR, Western Blotting or immunohistochemistry. Among these sodium transporters, the downregulation of NHE3, NCC, and NKCC2 was partially reversed by MnTBAP treatment. In contrast, the reduction of ENaCα was not affected by MnTBAP. The β and γ subunits of ENaC were not significantly altered by ureteral obstruction or MnTBAP therapy. To further confirm the anti-oxidant effect of MnTBAP, we examined the levels of TBARs in the urine collected from the obstructed ureters of UUO mice and bladder of sham mice. As expected, the increment of urinary TBARs in UUO mice was entirely abolished by MnTBAP therapy, indicating an amelioration of oxidative stress. Meantime, we found that three types of SOD were all reduced in obstructed kidneys determined by qRT-PCR, which was unaffected by MnTBAP. Collectively, these results demonstrated an important role of mitochondrial oxidative stress in mediating the downregulation of sodium transporters in obstructive kidney disease.

## INTRODUCTION

Unilateral ureteral obstruction (UUO) is a well-established experimental rodent model that mimics the complex pathophysiology of chronic obstructive nephropathy in an accelerated manner. Release of obstruction is often associated with diuresis or natriuresis, which is related to the downregulation of water channels and sodium transporters in obstructed kidneys [1]. It has been demonstrated that both Angiotensin (Ang) II [2] and cyclooxygenase-2 (COX-2)-derived prostaglandin E2 (PGE2) [3] mediated the decrease of sodium transporters in obstructive kidneys. However, the mechanisms mediating the downregulation of sodium transporters remain incompletely understood.

Oxidative stress contributes importantly to the pathogenesis of UUO [4–6]. ROS production in the kidney is derived from multiple intracellular mechanisms including xanthine oxidase, cytochrome P450 systems, uncoupled Nitric Oxide synthase (NOS), mitochondrial respiratory chain and nicotinamide adenine dinucleotide phosphate (NADPH) oxidase (NOXs) [7–9]. It has been shown that oxidative stress in UUO contributes to the development of tubulointerstitial lesions and renal fibrosis [7–9]. Liu *et al.* demonstrated that NOX-derived ROS are partially responsible for the downregulation of renal tubular Na/K-ATPase expression in acute UUO rats [10]. Our group reported that inhibition of mitochondrial complex-1 also reversed the downregulation of aquaporins [11] and sodium transporters including NKCC2 and ENaCα [12] in obstructive nephropathy. However, whether mitochondria-derived ROS is involved in the downregulation of sodium transporters during kidney obstruction is not known.

Therefore, in the present study, we employed manganese (III) tetrakis (4-benzoic acid) porphyrin chloride (MnTBAP), a synthetic mitochondrial superoxide dismutase 2 (SOD2) mimic, to investigate whether mitochondrial ROS could affect the downregulation of sodium transporters in the obstructed kidneys.

## RESULTS

### Effects of MnTBAP therapy on the protein expressions of NHE3 and NCC in obstructed kidneys

**Figure 1:**
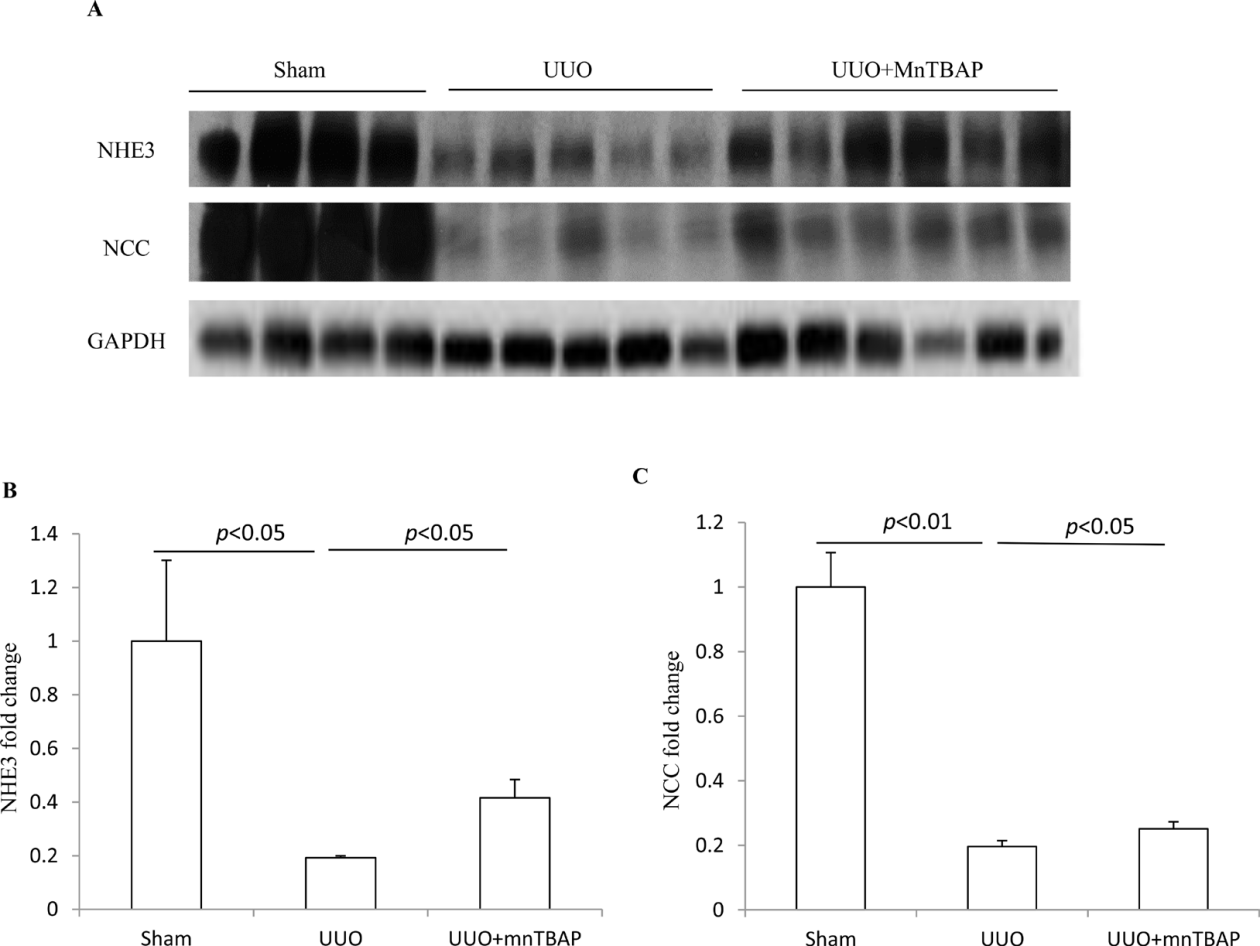
Protein expressions of NHE3 and NCC in obstructed kidneys following MnTBAP treatment (**A**) Western blotting analysis of NHE3 and NCC. (**B**) Densitometric analysis of NHE3 normalized by GAPDH. (**C**) Densitometric analysis of NCC normalized by GAPDH. The presented data are means ± SE. *N* = 6 in each group.

To detect the effect of MnTBAP therapy on the expression of sodium transporters in obstructed kidneys, we firstly examined the protein levels of NHE3 and NCC using Western blotting. As shown in Figure 1A–1C, both NHE3 and NCC protein expression were robustly downregulated in obstructive kidneys while MnTBAP therapy resulted in partially but significantly reversed the downregulation of NHE3 and NCC. These data indicated that mitochondrial oxidative stress was possibly involved in the downregulation of NHE3 and NCC in obstructed kidneys.

### Effect of MnTBAP therapy on the protein expression of NKCC2 in obstructed kidneys

**Figure 2:**
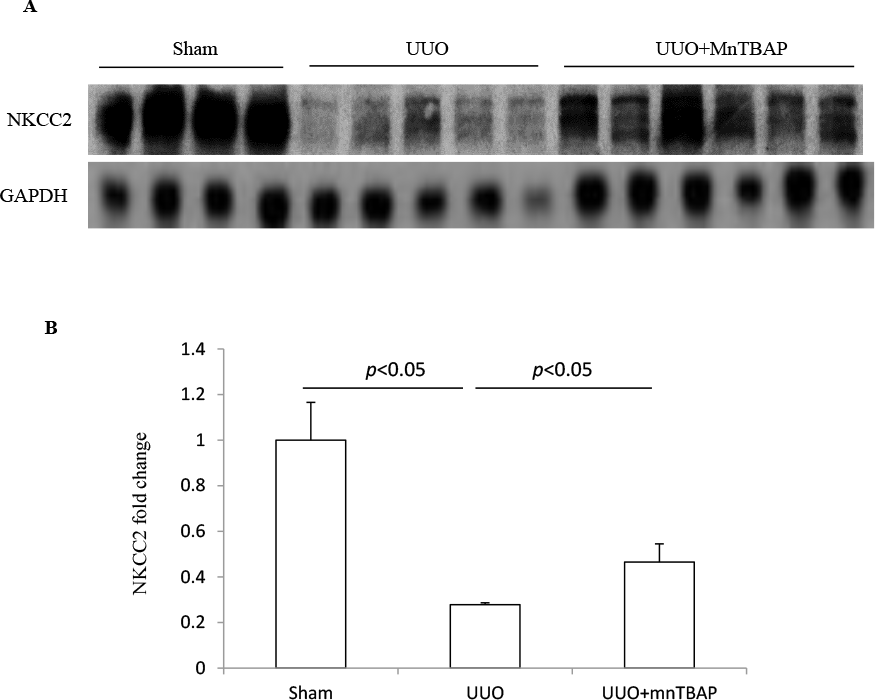
Protein expression of NKCC2 in obstructed kidneys following MnTBAP treatment (**A**) Western blotting analysis of NKCC2. (**B**) Densitometric analysis of NKCC2 normalized by GAPDH. The presented data are means ± SE. *N* = 6 in each group.

**Figure 3:**
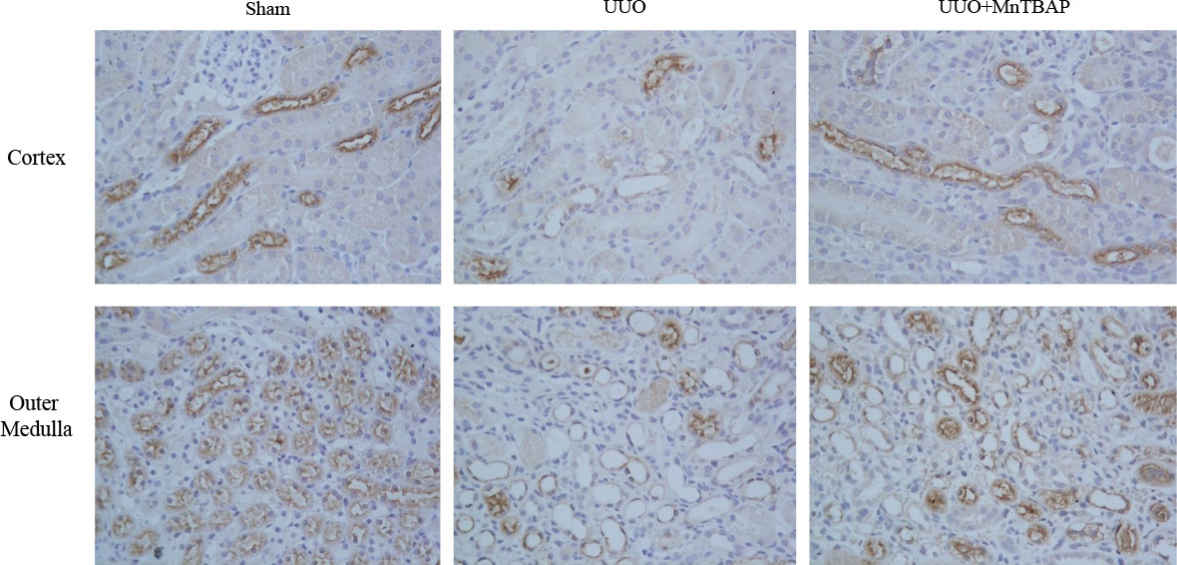
Immunohistochemistry of NKCC2 in obstructed kidneys following MnTBAP treatment

Next, we detected the effect of MnTBAP therapy on the protein expression of NKCC2. Consistent with previous reports [1, 12, 13], NKCC2 protein expression in obstructed kidneys was dramatically decreased. Upon administration of MnTBAP, the reduction was significantly ameliorated (Figure 2A, 2B). By immunochemistry, we further confirmed the change of NKCC2 in obstructed kidneys with or without MnTBAP treatment (Figure 3). These data also suggested an important role of mitochondrial ROS in mediating the downregulation of NKCC2 in the obstructed kidneys.

### Effects of MnTBAP on the protein expressions of ENaC subunits in obstructed kidneys

**Figure 4:**
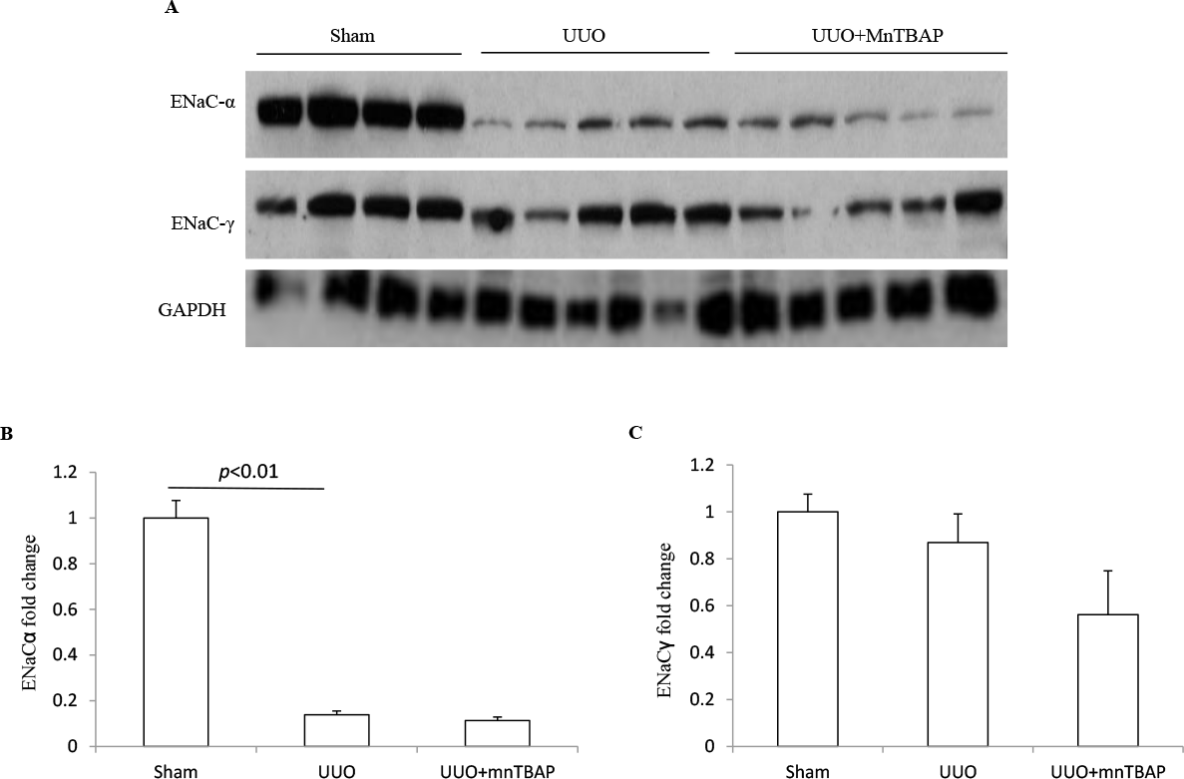
Protein expressions of ENaCα and ENaCγ in obstructed kidneys following MnTBAP treatment (**A**) Western blotting analysis of ENaCα and ENaCγ. (**B**) Densitometric analysis of ENaCα normalized by GAPDH. (**C**) Densitometric analysis of ENaCγ normalized by GAPDH. The presented data are means ± SE. *N* = 6 in each group.

The epithelial sodium channel(ENaC) is expressed in the aldosterone sensitive distal nephron(ASDN) and consists of 3 subunits. In previous studies, we found distinct changes of three ENaC subunits in obstructed kidneys [12]. Here, similar to the sodium transporters described above, the protein level of ENaCα displayed a marked downregulation in obstructed kidneys. However, MnTBAP administration did not affect the reduction of ENaCα in obstructed kidneys (Figure 4A, 4B). For ENaCγ, we observed a trend of downregulation in obstructed kidneys, which was not altered by MnTBAP therapy (Figure 4A, 4C). These results suggested that the alteration of ENaC subunits in obstructed kidneys is possibly independent of mitochondrial oxidative stress.

### Effects of MnTBAP on the mRNA expressions of sodium transporters in obstructed kidneys

**Figure 5:**
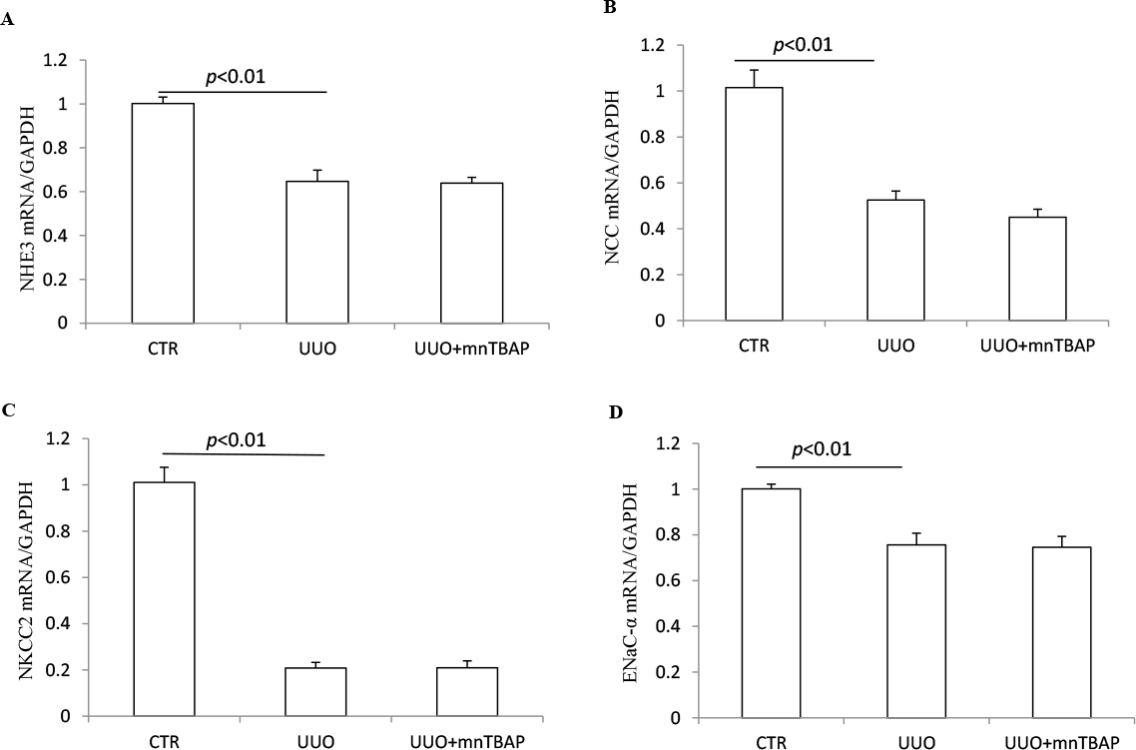
mRNA expressions of sodium transporters in obstructed kidneys following MnTBAP treatment (**A**) qRT-PCR analysis of NHE3. (**B**) qRT-PCR analysis of NCC. (**C**) qRT-PCR analysis of NKCC2. (**D**) qRT-PCR analysis of ENaCα. The presented data are means ± SE. *N* = 6 in each group.

To clarify whether MnTBAP therapy affected the regulation of sodium transporters at a transcriptional level, we analyzed the mRNA expressions of individual transporters via qRT-PCR. In obstructed kidneys, NHE3, NCC, NKCC2, and ENaCα were all significantly downregulated (Figure 5A–5D). However, none of the decreased transporters was influenced by MnTBAP therapy (Figure 5A–5D). These results implied that mitochondrial oxidative stress might be involved in the downregulation of the protein levels of sodium transporters along the nephron except ENaCs via a posttranscriptional mechanism. Thus, inhibition of the mitochondrial oxidative stress by MnTBAP couldn’t rescue the downregulation of sodium transporters at a transcriptional level, leading to a partial effect of MnTBAP therapy on the reduced sodium transporters of NHE3, NCC, and NKCC2.

### Effects of MnTBAP on the expressions of SODs and oxidative stress in obstructed kidneys

**Figure 6:**
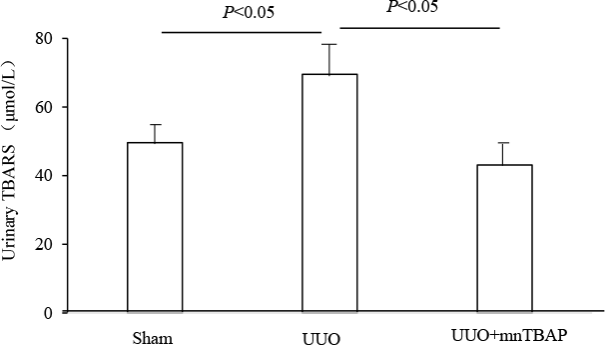
Urinary TBARS levels in UUO mice following MnTBAP treatment The presented data are means ± SE. *N* = 6 in each group.

**Figure 7:**
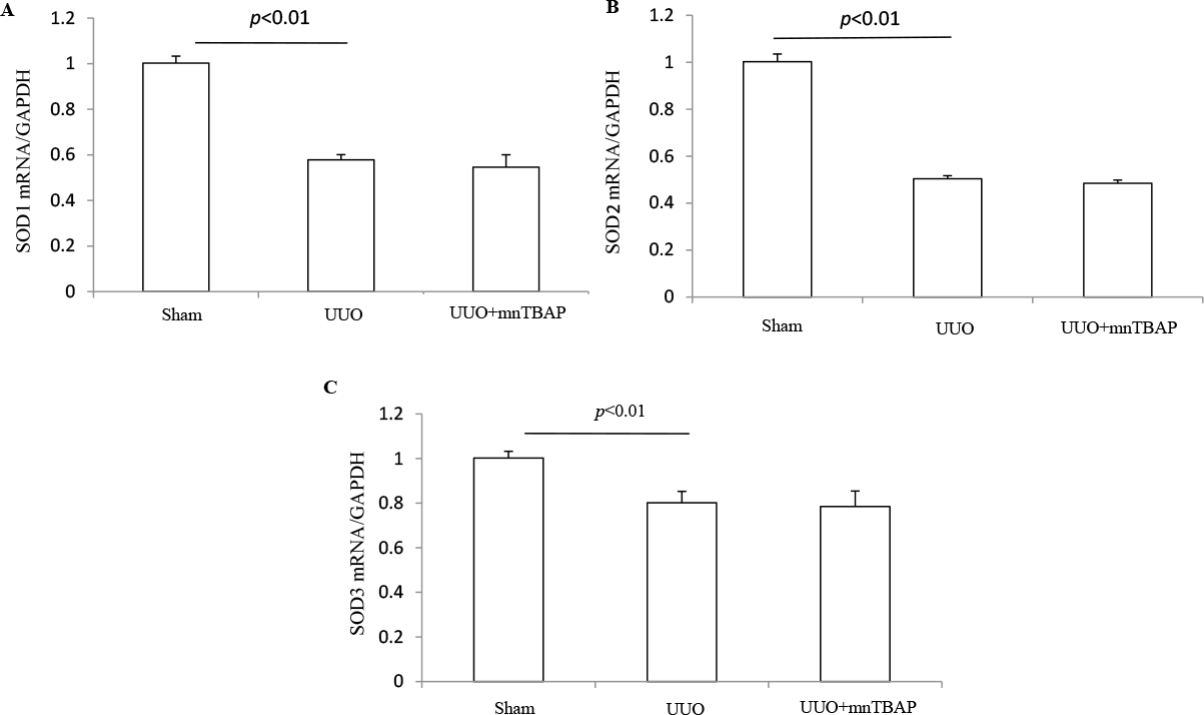
mRNA expressions of SOD1-3 in obstructed kidneys following MnTBAP treatment (**A**) qRT-PCR analysis of SOD1. (**B**) qRT-PCR analysis of SOD2. (**C**) qRT-PCR analysis of SOD3. The presented data are means ± SE. *N* = 6 in each group.

**Figure 8:**
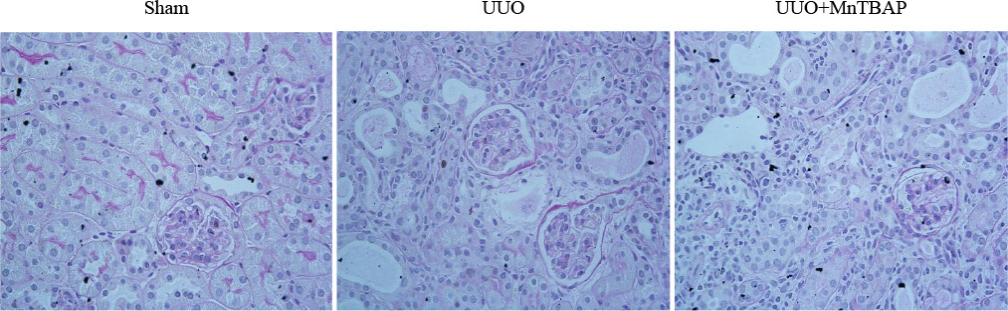
PAS staining in obstructed kidneys following MnTBAP treatment

As a mitochondrial SOD mimics, MnTBAP showed a protective role against the downregulation of sodium transporters in obstructed kidneys, suggesting a possible defect of SOD-related antioxidant system. To validate the anti-oxidative effect of MnTBAP in this model, we measured the urinary levels of TBARS, a known marker of oxidative stress, using the urine collected from the obstructed ureters of UUO mice and bladder of sham mice. As shown in Figure 6, MnTBAP therapy completely normalized the increment of TBARS concentration in urine. Furthermore, we analyzed the profiles of SODs (SOD1, SOD2, and SOD3) by qRT-PCR. Notably, all three forms of SODs were significantly downregulated in obstructed kidneys, which was not affected by MnTBAP therapy (Figure 7A–7C). These results suggested a potent role of MnTBAP in antagonizing the oxidative stress in obstructive kidneys. Finally, we detected the morphological changes using a PAS staining and found that MnTBAP treatment did not significantly affected the tubular structure in obstructed kidneys (Figure 8).

## DISCUSSION

Urinary tract obstruction is a serious disorder accompanied with impaired renal function such as a compromised ability to regulate the urinary excretion of water and sodium. It has been well documented that the abundance of sodium transporters was significantly reduced in response to ureteral obstruction, which at least partially contribute to the impaired urine concentrating capacity after the release of urinary tract obstruction [1, 13–15]. Consistent with previous finding, our data showed that the renal expression of sodium transporters including NHE3, NCC, NKCC2, and ENaCα were robustly decreased after ureteral obstruction while the expression of ENaCγ was not significantly changed in obstructed kidneys. Unlike other sodium transporters, the distinct regulation of ENaC subunits was reported previously by our and other groups [12, 16, 17].

Oxidative stress was well described to contribute importantly to the pathogenesis of obstructive nephropathy [6, 18–22]. The hydrostatic pressure and ischemia decreased intrarenal oxygen tension and resulted in increased oxidative stress. Various ROS sources implicated in renal diseases include the mitochondrial respiratory chain [23], NADPH oxidase [24, 25], xanthine oxidase [26], cyclooxygenase [27], lipoxygenase and uncoupled nitric oxide synthase (NOS) [28]. Among them, mitochondria are regarded as the major source of ROS since they are the primary intracellular sites of oxygen consumption. Xu *et al.* demonstrated that mitochondrial oxidative stress was associated with autophagy and apoptosis in tubular cells and represented an important mechanism of tubular decomposition and nephron loss during UUO [29]. Nishida’s group reported that mitochondrial dysfunction led to an elevation of ROS, which resulted in kidney fibrosis by stimulating cellular transformation to myofibroblasts during obstructive nephropathy [30]. Moreover, Manucha *et al.* reviewed that there existed an interaction among mitochondrial oxidative stress, apoptosis and inflammation during obstructive nephropathy [19]. Our group further confirmed this interaction by showing that mitochondrial complex I inhibitor rotenone attenuated not only oxidative stress but also inflammation and fibrosis in chronic obstructive uropathy [31]. At the meantime, we observed attenuated downregulation of sodium transporters by rotenone in UUO mice, which indicated the involvement of mitochondria dysfunction in defective sodium handling in obstructed kidney. Mitochondrial dysfunction results in ATP depletion, reactive oxygen species (ROS) overproduction, and the release of proapoptotic factors such as cytochrome C and mitochondrial DNA. The known role of mitochondria-localized SOD2 in antagonizing oxidative stress gave us a clue that mitochondrial oxidative stress might contributed to the dysregulation of sodium transporter during UUO. The superoxide dismutase (SOD) family is the main antioxidant system and is of importance in oxidative stress modulation [32]. Increased oxidative stress in obstructive nephropathy is always accompanied by decreased SOD expression [5, 33]. Our results demonstrated that the mRNA levels of SOD1-3 were significantly diminished in obstructed kidneys. Hence, we administered a SOD mimetic compound, MnTBAP, to the mice after the surgery of UUO. Not surprisingly, the mice given MnTBAP exhibited partial but significant amelioration of the downregulation of NHE3, NCC, NKCC2. However, the downregulation of ENaCα protein was not affected by MnTBAP therapy, suggesting that mitochondrial oxidative stress might not be important in modulating the sodium channels in collecting duct.

Another finding is that MnTBAP has impact only on the protein but not mRNA levels of NHE3, NCC, and NKCC2. This suggested that mitochondrial ROS may regulate these three transporters at posttranscriptional level. However, the exact mechanism mediating such an effect is still unknown. Then, we detected the expressions of SOD1-3 after kidney obstruction and MnTBAP administration. MnTBAP treatment normalized the oxidative stress marker TBARS in urine but did not affect the reduction of SOD1-3 during kidney obstruction, which might suggest that MnTBAP administration ameliorated oxidative stress during obstructive nephropathy without affecting the endogenous SOD.

Taken together, our study showed that the mitochondrial SOD mimics MnTBAP partially but significantly attenuated obstruction-induced downregulation of NHE3, NCC, and NKCC2 at protein levels in kidneys. The reduced mRNA levels of these transporters were not influenced by MnTBAP therapy. Meanwhile, MnTBAP did not affect the expressions of ENaC subunits in UUO model. These findings suggested that mitochondrial oxidative stress might mediate the downregulation of sodium transporters except ENaC in obstructive nephropathy via a posttranscriptional mechanism. This study not only provided new insights into the understanding of the pathogenesis of obstructive nephropathy-related dysregulation of sodium transporters but also offered new therapeutic target for the management of obstructive nephropathy-related fluid imbalance.

## MATERIALS AND METHODS

### Animals

C57BL/6J mice were originally purchased from the Jackson laboratory. This mouse colony was propagated at the Nanjing Medical University. In all studies, 3- to 4-month-old male mice were used. All mice were maintained under a 12: 12 h light-dark cycle (lights on at 6:00 a.m. and lights off at 6:00 p.m.). All experiments were approved by the Institutional Animal Care and Use Committee of Nanjing Medical University.

### Establishment of UUO mouse model and MnTBAP therapy

Unilateral ureteral obstruction (UUO) was induced as described previously [12, 31, 34]. Briefly, the left ureter was exposed and subsequently ligated with 6.0 silk through a small abdominal incision under the anesthesia with 2.0% isoflurane. The abdomen was closed in two layers. All mice received analgesia (subcutaneous injection of 50 μg/kg buprenorphine (Temgesic, Schering-Plough)) after the surgery. Following the surgery, the UUO mice were treated with Manganese (III) tetrakis (4-benzoic acid) porphyrin chloride (MnTBAP) (Catalog No.: sc-221954A, Santa Cruz, Dallas, TA, USA), at a dose of 10mg/kg/day in water by intraperitoneal injection. The control mice received sham operation and were treated with the same volume of water. All mice (*N* = 6 per group) were sacrificed 7 days after operation and the kidney tissues were harvested for further analysis.

### Immunohistochemistry

The kidneys were fixed in 10% formalin and embedded in paraffin. Kidney sections (4-μm thickness) were deparaffinized, hydrated in ethyl alcohol and washed in tap water. Endogenous peroxidase activity was blocked in 3% H_2_O_2_ for 15 min. Antigen retrieval was carried out in boiling antigen retrieval solution (1mmol/L Tris-HCl, 0.1mmol/L EDTA, pH = 8.0) for 15minutes. Then the sections were incubated overnight at 4°C with rabbit anti-NKCC2 antibody (Catalog No.: SPC-401, Stressmarq Biosciences Inc., Canada) and at room temperature for 30 min with secondary antibody. Staining was visualized using an ABC kit (Santa Cruz Biotechnology).

### Immunoblotting

Proteins were extracted from the whole kidney and homogenized in protein lysis buffer. Equal amount of protein (60 μg) was subjected to SDS-polyacrylamide gel electrophoresis, and transferred to nitrocellulose membranes. The blots were blocked at room temperature (RT) for 1h with 5% nonfat dry milk in Tris-buffered saline (TBS) and probed with the following antibodies: rabbit anti-NHE3 (Catalog No.: AB3085, Abcam, Cambridge, MA), anti-NKCC2 (Catalog No.: SPC-401, Stressmarq Biosciences Inc., Canada), anti-NCC (Catalog No.: SPC-402, Stressmarq Biosciences Inc., Canada), anti-ENaCα (Catalog No.: SPC-403, Stressmarq Biosciences Inc., Canada) and anti-ENaCγ (Catalog No.: SPC-405, Stressmarq Biosciences Inc., Canada) at a dilution of 1: 1,000 at 4°C overnight. After incubated with secondary antibodies (Catalog No.: sc-2004, goat anti-rabbit IgG, Santa Cruz) at RT for 1h, membranes were visualized using Pierce™ ECL Western Blotting Substrate (Thermo Scientific). Quantification was performed using Image-Pro Plus 6.0 software.

### Quantitative real-time PCR (qRT-PCR)

Total RNA was isolated by using TRIzol (Invitrogen) and first-strand cDNA was synthesized from total RNA(4ug) using Superscript TM III first strand synthesis kit (Invitrogen). Quantitative PCR (qPCR) was performed using the SYBR Green Master Mix (Applied Biosystems, Warrington, UK) and the ABI 7500 Real-Time PCR Detection System (Applied Biosystems, Foster City). Oligonucleotides were designed using Primer3 software (available at http://frodo.wi.mit.edu/primer3/) and the sequences are listed in Table 1. Cycling conditions were 95°C for 10 min, followed by 40 repeats of 95°C for 15 s, and 60°C for 1 min. Data were normalized to housekeeping gene GAPDH and presented as fold increase compared with RNA isolated from sham kidney using the 2-^Δ Δ^ CT method.

**Table 1:** Sequences of qRT-PCR primers

Gene	Primer sequence	Accession number
GAPDH	5′-GTCTTCACTACCATGGAGAAGG-3′ 5′-TCATGGATGACCTTGGCCAG-3′	M32599
ENaCα	5′-GCTTCATCTTTACCTGTCGTTTC-3′ 5′-CCAGAGATTGGAGTTGTTCTTGT-3′	NM_011324
NCC	5′-GACAGGCACCAACAGTGAGA-3′ 5′-TAGAGATGGCGGAGATGGAG-3′	U61085
NKCC2	5′-GCTCTTCATTCGCCTCTCCT-3′ 5′-AGCCTATTGACCCACCGAAC-3′	NM_011389
NHE3	5′-CTGAGGAGGAACCGAGCA-3′ 5′-AGGCCCAGAACGATGAGTAG-3′	XM_993032
SOD1	5′-AAGGCCGTGTGCGTGCTGAA-3′ 5′-CAGGTCTCCAACATGCCTCT-3′	NM 921076
SOD2	5′-CGGCCTACGTGAACAATCTC-3′ 5′-GATAGCCTCCAGCAACTCTCC-3′	NM 013671
SOD3	5′-TTCTTGTTCTACGGCTTGCTAC-3′ 5′-CTCCATCCAGATCTCCAGCACT-3′	NM 011435

### Measurement of TBARS

The measurement of urinary thiobarbituric acid-reactive substances (TBARS) was based on the formation of malondialdehyde by using a commercially available TBARS Assay Kit (Catalog No.: 10009055, Cayman Chemical) according to the manufacturer’s instructions.

### Statistical analysis

All results were presented as means ± SE. The statistical analysis was performed using ANOVA followed by Bonferroni’s test with SPSS 13 statistical software. *p* < 0.05 was considered significant.
